# NaCl cotransporter abundance in urinary vesicles is increased by calcineurin inhibitors and predicts thiazide sensitivity

**DOI:** 10.1371/journal.pone.0176220

**Published:** 2017-04-21

**Authors:** Omar A. Z. Tutakhel, Arthur D. Moes, Marco A. Valdez-Flores, Marleen L. A. Kortenoeven, Mathijs v. D. Vrie, Sabina Jeleń, Robert A. Fenton, Robert Zietse, Joost G. J. Hoenderop, Ewout J. Hoorn, Luuk Hilbrands, René J. M. Bindels

**Affiliations:** 1 Department of Physiology, Radboud University Medical Center, Radboud Institute for Molecular Life Sciences, Nijmegen, The Netherlands; 2 Department of Internal Medicine, Nephrology and Transplantation, Erasmus Medical Center, Rotterdam, The Netherlands; 3 Programa Regional en Doctorado en Biotecnología, Universidad Autónoma de Sinaloa, Sinaloa, Mexico; 4 Department of Biomedicine, Center for Interaction of Proteins in Epithelial Transport, Aarhus University, Aarhus, Denmark; 5 Department of Nephrology, Radboud university medical center, Radboud Institute for Molecular Life Sciences, Nijmegen, The Netherlands; University Medical Center Utrecht, NETHERLANDS

## Abstract

Animal studies have shown that the calcineurin inhibitors (CNIs) cyclosporine and tacrolimus can activate the thiazide-sensitive NaCl cotransporter (NCC). A common side effect of CNIs is hypertension. Renal salt transporters such as NCC are excreted in urinary extracellular vesicles (uEVs) after internalization into multivesicular bodies. Human studies indicate that CNIs also increase NCC abundance in uEVs, but results are conflicting and no relationship with NCC function has been shown. Therefore, we investigated the effects of CsA and Tac on the abundance of both total NCC (tNCC) and phosphorylated NCC at Thr60 phosphorylation site (pNCC) in uEVs, and assessed whether NCC abundance in uEVs predicts the blood pressure response to thiazide diuretics. Our results show that in kidney transplant recipients treated with cyclosporine (n = 9) or tacrolimus (n = 23), the abundance of both tNCC and pNCC in uEVs is 4–5 fold higher than in CNI-free kidney transplant recipients (n = 13) or healthy volunteers (n = 6). In hypertensive kidney transplant recipients, higher abundances of tNCC and pNCC prior to treatment with thiazides predicted the blood pressure response to thiazides. During thiazide treatment, the abundance of pNCC in uEVs increased in responders (n = 10), but markedly decreased in non-responders (n = 8). Thus, our results show that CNIs increase the abundance of both tNCC and pNCC in uEVs, and these increases correlate with the blood pressure response to thiazides. This implies that assessment of NCC in uEVs could represent an alternate method to guide anti-hypertensive therapy in kidney transplant recipients.

## Introduction

The calcineurin inhibitors (CNIs) cyclosporine A (CsA) and tacrolimus (Tac) are widely used to prevent rejection of transplanted organs. CNIs inhibit the calcineurin-mediated immune response in T-cells [1]. Although both CsA and Tac exert their principal immunosuppressive effects through inhibition of the same target protein, calcineurin, they differ in cytoplasmic-binding proteins, namely cyclophilins and FKBP12 for CsA and Tac, respectively. CsA and Tac also vary with respect to their immunosuppressive potency [2,3] and side effects [4–6]. A common side effect of CNIs is hypertension, although CsA appears more hypertensinogenic than Tac [6–8]. CNI-induced hypertension may be accompanied by hyperkalemia and metabolic acidosis [9,10]. The clinical characteristics of CNI-treated patients sometimes resemble that of familial hyperkalemic hypertension (FHHt) [11,12], also known as Gordon syndrome [13] or pseudohypoaldosteronism type II [14] (OMIM 145260). FHHt results from mutations in WNK [with no lysine (K)] kinases WNK1 and WNK4 [15], Kelch-like 3 (KLHL3) [16], or Cullin 3 (CUL3) [17], which all lead to a gain-of-function in the thiazide-sensitive NaCl cotransporter (NCC) resulting in salt retention in the distal part of the nephron [15,18–20]. Several studies have shown that CNIs increase NCC activity possibly contributing to hypertension [21,22]. Melnikov *et al*. demonstrated that rats treated with CsA develop a phenotype similar to that of FHHt, which they attributed to an increase in WNK4 abundance in the kidney [23]. This phenomenon is supported by *in vitro* studies showing that the abundance of WNK4 and ultimately of total NCC (tNCC) and phosphorylated, or active, NCC (pNCC), is increased in immortalized mouse distal convoluted tubule (mDCT) cells treated with CsA [23]. Hoorn *et al*. revealed that Tac-induced hypertension in mice is predominantly mediated by an increase in pNCC abundance, possibly through an effect of the NCC-regulating kinases WNK3, WNK4, and STE20/SPS1-related proline/alanine-rich kinase (SPAK) [21]. Recent evidence suggests that in mice, Tac prevents the high potassium stimulated NCC dephosphorylation [24]. Additionally, it has been demonstrated that Tac acts directly on kidney tubule cells expressing NCC to cause hypertension, and that inhibition of calcineurin is required for this effect [22].

In humans, urinary extracellular vesicles (uEVs), including exosomes, have been extensively characterized and studied as non-invasive biomarkers for renal tubular disorders [25–28]. uEVs are nanosized membranous vesicles released from all cells lining the nephron. Alterations in the expression of different proteins present in the epithelial cells of the distal convoluted tubule (DCT), including NCC, are reflected in the composition of uEVs [29–32]. Patients with FHHt have an increased NCC abundance in uEVs [31–33], while patients with Gitelman syndrome exhibit a decreased NCC abundance in uEVs [28,34]. In humans, mineralocorticoid administration rapidly increased the abundance of tNCC and pNCC in uEVs [35], possibly mediated by a reduced plasma potassium concentration secondary to epithelial sodium channel (ENaC) activation [35,36]. This suggests that NCC abundance in uEVs reflects the actual state of NCC expression in the epithelial cells of the DCT. Accordingly, we previously showed that in patients with primary aldosteronism, pNCC increased similarly in kidney and uEVs [31].

Although protein abundance and characterization in uEVs can potentially be used as biomarkers for some diseases [28,31–34], only a few studies have been conducted to investigate the role of NCC in CNI-induced hypertension in humans. Esteva-Font *et al*. found a positive correlation between plasma CsA levels and NCC abundance in uEVs of CsA-treated kidney transplant recipients [37]. Rojas-Vega *et al*. showed an increased abundance of NCC in uEVs of Tac-treated hypertensive kidney transplant recipients [38]. Although these studies showed the stimulatory effect of CNIs on NCC abundance in uEVs of kidney transplant recipients, they did not explore the relationship between NCC abundance and blood pressure in kidney transplant recipients. Therefore, in the present study, we performed a large-scale study to investigate the effect of CsA and Tac on the abundance of both tNCC and pNCC in uEVs, and assessed whether NCC abundance in uEVs predicts the blood pressure response to thiazide diuretics. Finally, in order to confirm the effect of CNIs on NCC in the kidney, an *ex vivo* study was conducted in mice cortical tubules exposed to CsA.

## Materials and methods

### Study design and population

Two groups of kidney transplant recipients using CNIs were studied. Group 1 was recruited at the Radboud university medical center, in Nijmegen, The Netherlands, and consisted of a randomly selected cohort of 45 kidney transplant recipients and 6 healthy volunteers of whom uEVs were isolated and analyzed. The kidney transplant recipients used CsA (n = 9), Tac (n = 23) or a CNI-free immunosuppressive regimen (n = 13) for at least 6 months and were matched for age and gender. Kidney transplant recipients who had been using thiazide diuretics or aldosterone antagonists after transplantation were excluded. Group 2 consisted of Tac-treated hypertensive kidney transplant recipients (median of 2.4 years after kidney transplantation), recruited from a clinical trial studying the anti-hypertensive effect of thiazide-type diuretic chlorthalidone at the Erasmus Medical Center, in Rotterdam, The Netherlands [39]. Patients with an office blood pressure >140/90 mmHg were invited for ambulatory blood pressure measurement. In this group, 18 patients with an average daytime systolic blood pressure >140 mmHg were enrolled and followed for 8 weeks chlorthalidone (12–25 mg once daily) treatment. Patients who responded to chlorthalidone (‘responders’, decrease of ≥10 mmHg in average daytime systolic blood pressure, n = 10) were compared with patients who did not respond to chlorthalidone (‘non-responders’, no change or an increase in average daytime systolic blood pressure, n = 8). All participants gave written informed consent and both cohorts were approved by Medical Ethics Committee (CMO09/073 for Radboud university medical center and MEC-2012-417 for Erasmus Medical Center) and this study was conducted according to the principles expressed in the Declaration of Helsinki.

### Urine collection and isolation of extracellular vesicles

In Group 1, second-morning mid-stream urine sample was collected. In Group 2, second-morning mid-stream urine was collected just before starting and after 8 weeks of chlorthalidone treatment. In both groups, immediately after urine collection, the protease inhibitors (50 μmol/L phenylmethylsulfonyl fluoride, 20 μmol/L aprotinin, 10 μmol/L pepstatin A, and 20 μmol/L leupeptin) were added to the urine to reduce protein degradation. All samples were directly stored at -80°C. uEVs were isolated as reported previously [29–31,40]. In brief, 10 to 40 mL of the collected urine samples were centrifuged at 17,000 × g for 15 minutes at 24°C in an ultracentrifuge (Sorvall^™^ WX Floor Ultra Centrifuges, Thermo Scientific, Asheville, NC, USA) with a 70.1Ti rotor. The supernatant was stored at room temperature for 25 minutes. The pellet was resuspended in 50 μL of 3.24 mol/L dithiothreitol and 200 μL isolation solution (10 mmol/L triethanolamine, 250 mmol/L sucrose, HCl pH 7.6) and centrifuged at 17,000 × g for 15 minutes at 24°C. Next, the supernatant was collected and combined with the supernatant obtained from the previous centrifugation, and the combined supernatants were centrifuged at 170,000 × g for 2.5 hours at 24°C. Pellets containing uEVs were solubilized in Laemmli sample buffer (0.6% w/v SDS, 3% v/v glycerol, 18 mmol/L Tris-HCl pH 6.8 and 0.003% w/v bromophenol blue). All the samples were preheated for 15 minutes at 65°C before immunoblotting. Urinary creatinine was measured according to Jaffe’s method with the use of a colorimetric assay (Labor und Technik, Berlin, Germany).

### Mouse cortical tubule suspension

Animal protocols were approved by the board of the Institute of Biomedicine, University of Aarhus. The animal protocols comply with the European Community guidelines for the use of experimental animals, were approved and performed under a license issued for the use of experimental animals by the Danish Ministry of Justice (Dyreforsøgstilsynet). For this study mice were anesthetized using isoflurane inhalation, followed by cervical dislocation. Both kidneys from a male wildtype C57BL/6J mouse were removed and dissected into approximately 1 mm pieces and placed into 4 mL of enzyme solution containing 1.5 mg/mL collagenase type B (Worthington Labs, Lakewood, NJ, USA) in basic buffer (125 mmol/L NaCl, 30 mmol/L glucose, 0.4 mmol/L KH_2_PO_4_, 1.6 mmol/L K_2_HPO_4_, 1 mmol/L MgSO_4_, 10 mmol/L Na-acetate, 1 mmol/L α-ketoglutarate, 1.3 mmol/L Ca-gluconate, 5 mmol/L glycine, 48 μg/mL trypsin inhibitor, and 50 μg/mL DNase, Tris-HCl pH 7.4). Samples were mixed continuously at 37°C using a benchtop orbital mixer. Samples were incubated for 15 minutes at 22°C to let the large fragments sink down to the bottom of the tube. Next, 2 mL of the enzyme solution was removed and replaced with 2 mL of basic buffer. After 10 minutes’ incubation at 4°C, an additional 2 mL of basic buffer was added, and samples were incubated for an additional 10 minutes at 22°C. Large fragments were allowed to settle, the supernatant was removed and centrifuged at 200 × g for 2 minutes. The pellet was resuspended in 5 mL of basic buffer (buffer B) (120 mmol/L NaCl, 30 mmol/L glucose, 1 mmol/L CaCl_2_, 1 mmol/L MgCl_2_, 1 mmol/L Na_2_HPO_4_, 1 mmol/L Na_2_SO_4_, 15 mmol/L Na-HEPES, Tris-HCl pH 7.4) and centrifuged at 200 × g for 2 minutes. The tubular suspensions were resuspended in buffer B and 500 μL was transferred into individual tubes containing either DMSO (negative control) or different concentrations of CsA (final concentrations of 5, 10, or 20 μmol/L). Hypotonic low chloride (buffer H), which stimulates NCC [41,42], was used as a positive control. Suspensions were incubated with continuous mixing for 30 and 90 minutes at 37°C. Tubules were centrifuged for 10 minutes at 3,000 × g at 4°C and pellets were resuspended in 300 μL Laemmli sample buffer containing dithiothreitol (50 mg/mL). Finally, the samples were heated for 15 minutes at 60°C before immunoblotting.

### Immunoblotting

uEV-samples were loaded on a gradient SDS-PAGE gel (4–15% v/v Criterion^™^ TGX^™^ Precast Gel, Bio-Rad, The Netherlands). Loading of uEV-sample of each subject was normalized to urinary creatinine concentration to account for uEV concentration differences between the individual samples [26]. Subsequently, immunoblotting was performed on polyvinylidene difluoride membranes (Immobilon-P, Millipore Corporation, Bedford, MA, USA), which were blocked and probed with antigen-specific primary antibodies. Blots were incubated with species-specific fluorescent secondary antibodies and visualized using enhanced chemiluminescence (Thermo Fischer Scientific, Waltham, MA, USA) and gel imaging system (ChemiDoc XRS, Bio-Rad Laboratories, Hercules, CA, USA). Finally, both the dimeric and monomeric forms of the NCC bands on the blots were quantified together with Image Studio Lite software (LI-COR Biosciences, NE, USA) or ImageQuant TL (GE Healthcare Life Sciences, PA, USA). To analyze whether normalization by urinary creatinine resulted in a similar number of uEVs loaded on a gel, the abundance of the uEV-marker CD9 was measured [26,30,43].

### Antibodies

The following antibodies were used: anti-total NCC (Millipore, Billerica, MA, USA, #AB3553; 1:2,000); anti-human phosphorylated NCC at Thr60 or (anti-mouse phosphorylated NCC at Thr58; 1:2,000) as previously described [44] and anti-uEV marker CD9 of human origin (C4, Santa Cruz Biotechnology, Inc., CA, USA; 1:500). Secondary antibodies used were peroxidase-conjugated goat anti-rabbit (Sigma-Aldrich, St. Louis, MO, USA; 1:10,000), and anti-mouse (Sigma-Aldrich, St. Louis, MO, USA; 1:10,000).

### Statistical analysis

Values are expressed as mean ± SEM. In Group 1 the immunoblot data were analyzed by comparing integrated optical densities of bands by one-way ANOVA with Dunnett multiple comparisons *post hoc* test. In Group 2 the immunoblot data were analyzed using a non-parametric Student's *t*-test (comparing responders to non-responders). Two-way ANOVA was used to assess changes in uEV protein abundance before and after the chlorthalidone treatment. In the mouse study the immunoblot data were analyzed by comparing integrated optical densities of bands after 30 and 90 minutes exposure to CsA separately compared to its negative control (basic buffer without CsA) by one-way ANOVA with Dunnett multiple comparisons *post hoc* test. In this study, fold-change of 1 means no change. *P*<0.05 was considered statistically significant. All data were analyzed using Prism 5 software (GraphPad Software Inc, La Jolla, CA, USA).

## Results

### CNIs increase NCC in uEVs of kidney transplant recipients

**Fig 1:**
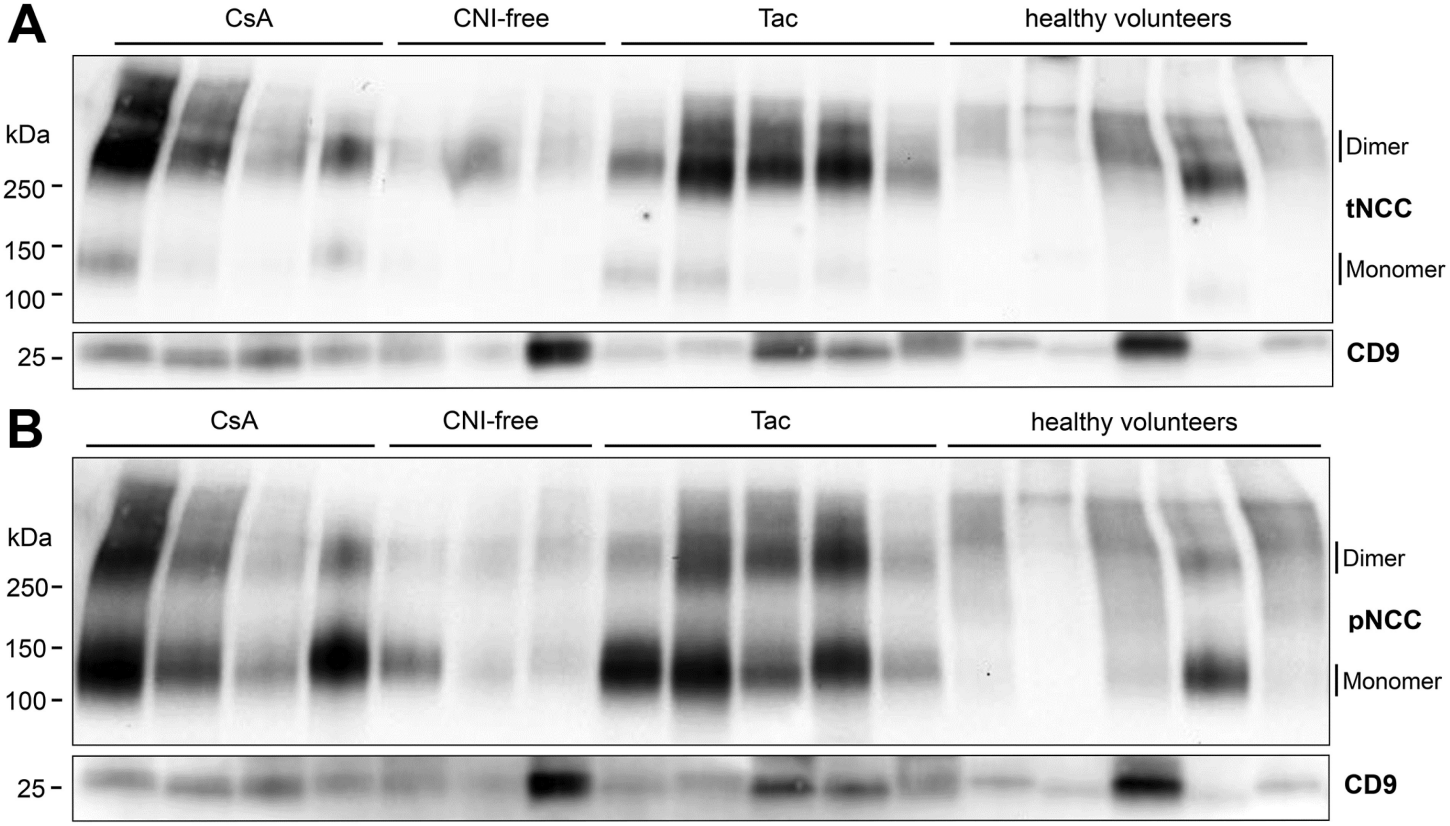
Representative immunoblots of tNCC and pNCC abundance in uEVs of kidney transplant recipients treated with CsA, Tac or CNI-free immunosuppressive regimens and healthy volunteers. Panels A and B show the immunoreactive bands in uEVs of patients treated with CsA (n = 4), Tac (n = 5), CNI-free immunosuppressive regimens (n = 3), and healthy volunteers (n = 5). tNCC (A) and pNCC (B) immunoreactive bands in uEVs of both CsA- and Tac-treated kidney transplant recipients were more abundant compared to kidney transplant recipients treated with CNI-free immunosuppressive regimens and healthy volunteers.

**Fig 2:**
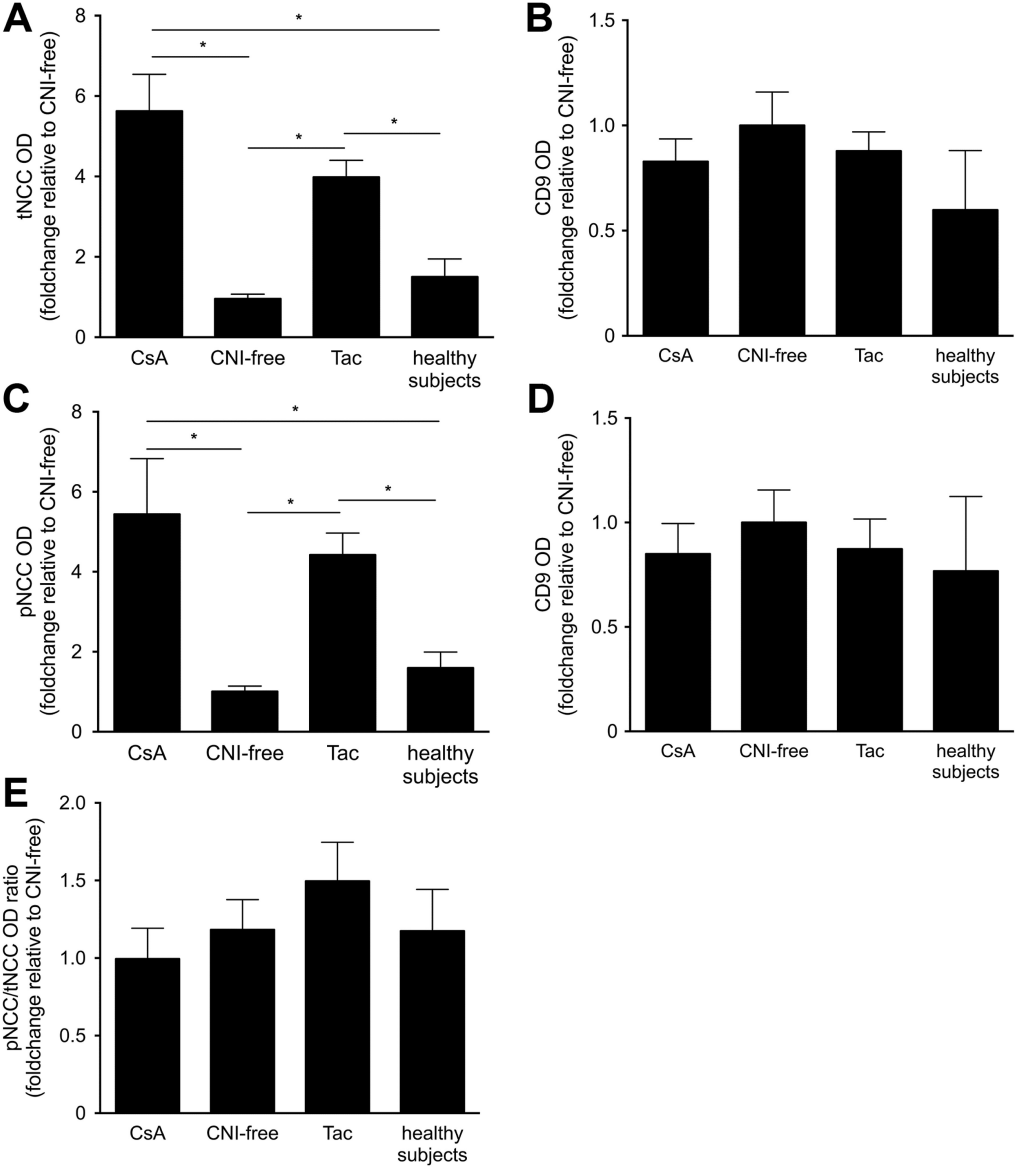
Densitometry of tNCC and pNCC immunoreactive bands in uEVs of all kidney transplant recipients treated with CsA (n = 9), Tac (n = 23) or CNI-free immunosuppressive regimens (n = 13) and healthy volunteers (n = 6). Both tNCC (A) and pNCC (C) abundance in both CsA- and Tac-treated kidney transplant recipients was significantly higher in comparison to kidney transplant recipients treated with CNI-free immunosuppressive regimens and healthy volunteers. Densitometry analysis of CD9 expression of the immunoblots for tNCC (B) and pNCC (D) showed no significant differences between the four groups. The ratio of pNCC to tNCC abundance in uEVs of CsA- and Tac-treated group was not significantly higher in comparison to kidney transplant recipients treated with CNI-free immunosuppressive regimens and healthy volunteers (E). The original immunoblots are shown in Fig 1 and S3 and S4 Figs. Values are mean ± SEM normalized to kidney transplant recipients treated with CNI-free immunosuppressive regimens (one-way ANOVA, *P<0.05, n = 51).

Table 1 shows the clinical and laboratory characteristics of 9 CsA, 23 Tac and 13 CNI-free immunosuppressive regimens treated kidney transplant recipients (Group 1). The effect of CNI on NCC abundance in uEVs was assessed using immunoblot analysis. Two immunoreactive bands of ~260 and ~130 kDa representing the dimeric and monomeric forms of the NCC protein were detected in uEVs (Fig 1). Both forms were included in the analysis to determine the effect of CNI treatment on NCC abundance in uEVs. The abundances of tNCC and pNCC in uEVs of both CsA and Tac-treated kidney transplant recipients were significantly higher compared to uEVs isolated from kidney transplant recipients treated with CNI-free immunosuppressive regimens or healthy volunteers (Fig 2A and 2C). No significant difference in tNCC and pNCC abundance was detected between CsA and Tac-treated kidney transplant recipients. The ratio of pNCC to tNCC did not differ between kidney transplant recipients treated with CsA, Tac, CNI-free immunosuppressive regimens, and healthy volunteers (Fig 2E). Females may express more NCC [45], but in our study a gender difference in the abundance of NCC in the uEVs was not demonstrated (S1 Fig). No significant differences in CD9 abundance were observed between the four experimental groups, suggesting comparable uEV numbers (Fig 2B and 2D). Additional normalization by CD9 abundance showed that both tNCC (S2A Fig) and pNCC (S2B Fig) abundance in both CsA- and Tac-treated kidney transplant recipients was significantly higher in comparison to kidney transplant recipients treated with CNI-free immunosuppressive regimens, but not healthy volunteers.

**Table 1:** Clinical and laboratory characteristics of the patients in study Group 1.

Characteristic (mean ± SEM)	CNI-free group (n = 13)	CsA group (n = 9)	Tac group (n = 23)	*P*-value
Age	53 ± 3	51 ± 2	52 ± 3	0.90^a^, 0.96^b^
Male (n (%))	7 (54)	5 (56)	12 (52)	
Body mass index, Kg/m^2^	25 ± 1	27 ± 2	27 ± 1	0.53^a^, 0.38^b^
Cause of ESRD (n)				
Polycystic kidney disease	3	1	5	
Diabetes	0	2	2	
Glomerular disease	2	0	0	
Hypertension/Vascular	0	0	2	
Other	8	6	14	
Plasma sodium, mmol/L	140 ± 1	138 ± 1	139 ± 1	0.44^a^, 0.72^b^
Plasma potassium, mmol/L	4.0 ± 0.1	4.1 ± 0.1	4.1 ± 0.1	0.80^a^, 0.72^b^
Plasma chloride, mmol/L	106 ± 1	105 ± 1	108 ± 1	0.80^a^, 0.29^b^
Plasma creatinine, mg/dL	1.6 ± 0.1	1.5 ± 0.1	1.7 ± 0.1	0.80^a^, 0.72^b^
eGFR, mL/min/1.73 m^2^	46 ± 2	42 ± 3	45 ± 4	0.76^a^, 0.97^b^
Systolic blood pressure, mmHg	135 ± 3	137 ± 4	142 ± 3	0.91^a^, 0.22^b^
Diastolic blood pressure, mmHg	80 ± 2	84 ± 4	82 ± 2	0.52^a^, 0.77^b^
CsA pre-dose level, μg/L		127 ± 14		
Tac pre-dose level, μg/L			6.7 ± 0.4	

SEM = standard error of the mean; ESRD = end stage renal disease; data are presented as mean ± SEM.

^*a*^ = *P*-value for difference between CsA and CNI-free;

^*b*^ = *P*-value for difference between Tac and CNI-free, 95% CI, 95% confidence interval.

### NCC abundance in uEVs predicts the anti-hypertensive response to thiazides

**Fig 3:**
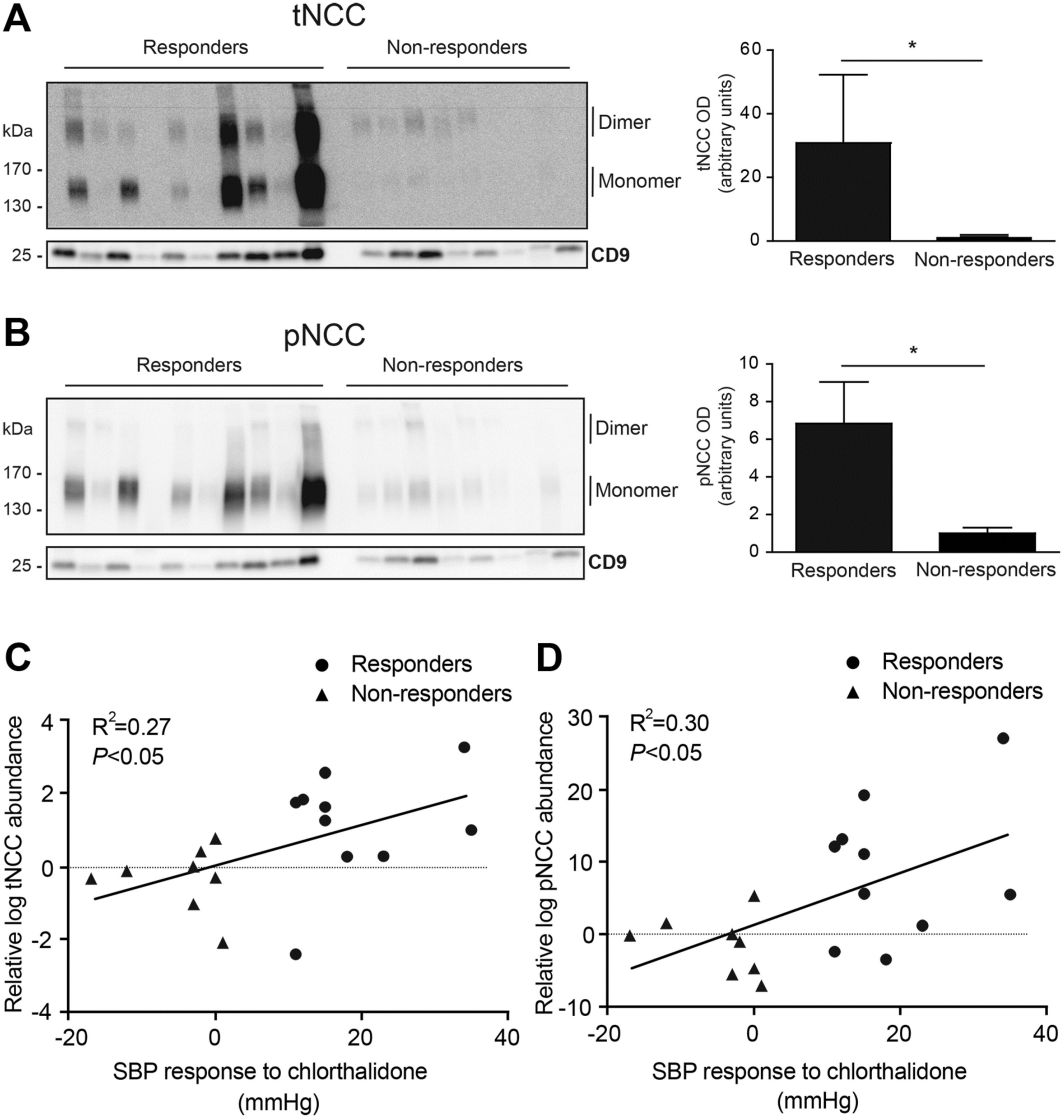
Pre-treatment tNCC and pNCC abundances in uEVs isolated from hypertensive kidney transplant recipients who did or did not respond to chlorthalidone. Shown are immunoblots of tNCC (panel A) and pNCC (panel B) together with CD9 in uEVs of hypertensive kidney transplant recipients using Tac. ‘Responders’ (n = 10) refer to patients who subsequently had a significant anti-hypertensive response (≥10 mmHg reduction in systolic blood pressure) to 8-week treatment with chlorthalidone. Non-responders (n = 8) did not have an anti-hypertensive response to chlorthalidone (no change or increase in systolic blood pressure). uEVs were isolated before the treatment with chlorthalidone. Both tNCC and pNCC abundance were significantly higher in responders compared to non-responders (non-parametric t-test, *P<0.05, n = 18). Both pNCC and tNCC abundance in uEVs correlated with the blood pressure response (panel C, R^2^ = 0.27 and panel D, R^2^ = 0.30 using log-transformed densitometry data because of non-normal distribution, *P*<0.05 for both). Abbreviations: SBP, ambulatory systolic blood pressure.

**Fig 4:**
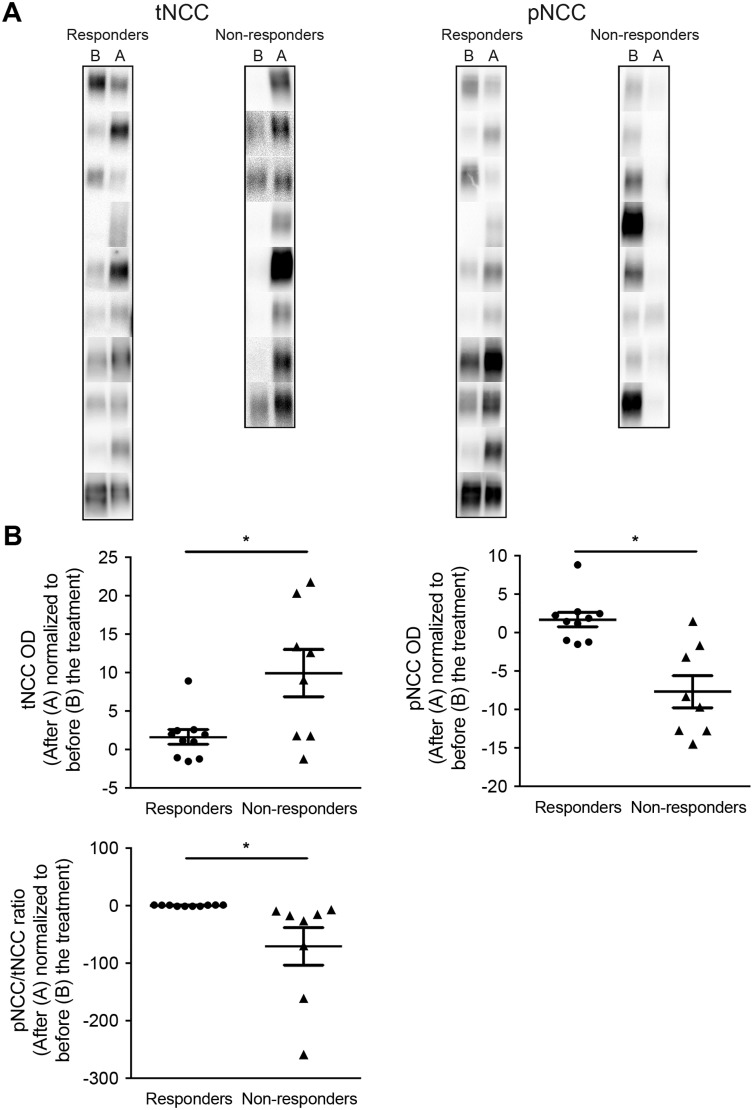
pNCC and tNCC abundances in uEVs before and after treatment with chlorthalidone. Panel A shows pNCC and tNCC abundances in uEVs before (B) and after (A) the 8-week treatment period with chlorthalidone in both responders (n = 10) and non-responders (n = 8). The fold-changes in the before-after abundances of pNCC and tNCC in uEVs (as measured by densitometry) of both responders and non-responders are shown in panel B (Fold-change of 1 means no change, *P<0.05). The scatter plots represent the fold change in tNCC, pNCC or their ratio after treatment with chlorthalidone (densitometry values before treatment with chlorthalidone were set to 1). S5 and S6 Figs, show the original immunoblots from which the individual panels in Fig 4A were derived.

Subsequently, we investigated whether the blood pressure response to thiazide diuretics in hypertensive kidney transplant recipients treated with Tac correlates with NCC abundance in uEVs. To this end, we compared tNCC and pNCC abundances in uEVs of patients with a blood pressure response to a thiazide in comparison to those who did not respond. Table 2 shows the clinical and laboratory characteristics of these patients (Group 2). Pre-treatment abundances of both tNCC and pNCC in uEVs were significantly higher in chlorthalidone responders compared to non-responders (Fig 3). Furthermore, both pNCC and tNCC abundance in uEVs correlated with the blood pressure response (R^2^ = 0.27 and 0.30 using log-transformed densitometry data because of non-normal distribution, *P*<0.05 for both, Fig 3). Subsequently, the change in NCC abundance before and after treatment with chlorthalidone in uEVs of both responders and non-responders was compared (Fig 4 and S5 and S6 Figs, show original immunoblots). The increase of tNCC in uEVs was larger in non-responders than in responders (*P*<0.05). While pNCC in uEVs also increased in most responders, there was a decrease in pNCC in the majority of non-responders (*P*<0.05). As a result, the pNCC to tNCC ratio remained constant in the responders, but markedly decreased in non-responders (*P*<0.05). This suggests that phospho-occupancy at the measured site was lower in non-responders. No gender differences were found (S7 Fig). Again, no significant differences were found in the abundances of CD9 between groups, suggesting comparable uEV-numbers (Fig 3 and S8 Fig). Additional normalization by CD9 abundance led to similar results (S9 Fig).

**Table 2:** Clinical and laboratory characteristics of the patients in study Group 2.

Characteristic (mean ± SEM)	Responders (n = 10)	Non-responders (n = 8)	*P*-value
Age	61 ± 2	57 ± 3	0.15
Males (n (%))	6 (60)	6 (75)	0.27
Body mass index, Kg/m^2^	27 ± 2	30 ± 2	0.14
Cause of ESRD (n)			
Polycystic kidney disease	1	0	
Diabetes	2	3	
Glomerular disease	0	3	
Hypertension/vascular	6	1	
Other	1	1	
Plasma sodium, mmol/L	140 ± 1	140 ± 1	0.40
Plasma potassium, mmol/L	4.5 ± 0.1	5.1 ± 0.2*	<0.01
Plasma chloride, mmol/L	103 ± 1	105 ± 1	0.07
Plasma creatinine, mg/dL	1.2 ± 0.1	1.7 ± 0.2*	0.01
eGFR, mL/min/1.73 m^2^	55 ± 5	43 ± 6	0.06
Urine K/creatinine, mmol/mmol	5.2 ± 2.1	4.6 ± 1.9	0.28
Systolic blood pressure, mmHg	158 ± 4	151 ± 5	0.15
Diastolic blood pressure, mmHg	86 ± 2	80 ± 3	0.07
Tac pre-dose level, μg/L	5.5 ± 0.4	5.8 ± 0.5	0.30

SEM = standard error of the mean; ESRD = end stage renal disease; data are presented as mean ± SEM. *P*-value for difference between responders and non-responders, 95% CI, 95% confidence interval.

### Increased pNCC in mouse cortical tubule suspensions exposed to CsA

**Fig 5:**
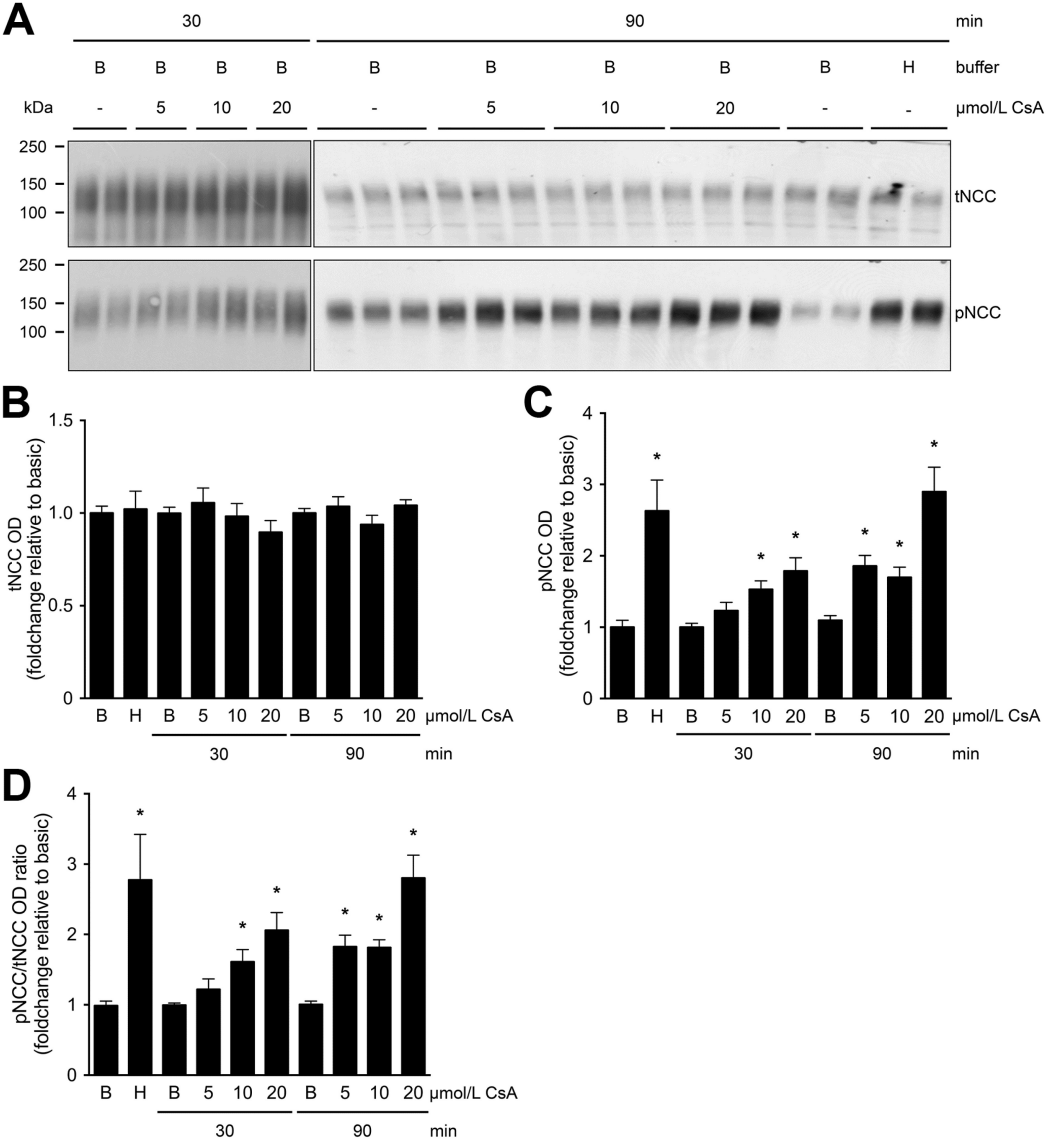
tNCC and pNCC abundance in mouse cortical tubule suspension exposed to CsA. Panel A shows representative immunoblots of tNCC and pNCC abundance in mouse cortical tubule suspension exposed to CsA. Immunoblots of protein homogenates of mouse cortical tubule suspensions were incubated in basic (B) buffer for 30 and 90 minutes, in the absence (-) or presence of CsA at final concentrations of 5, 10, or 20 μmol/L (A). tNCC remained at baseline levels (B), while pNCC (C) and ratio of pNCC to tNCC (D) were significantly increased in the mouse cortical tubule suspension after 30 minutes of exposure to 10 and 20 μmol/L of CsA and 90 minutes of exposure to 5, 10, and 20 μmol/L of CsA. Similarly, pNCC and the ratio of pNCC to tNCC were significantly increased in hypotonic low chloride (H) buffer (C and D). The original immunoblots for 30 and 90 minutes, in the absence (-) or presence of CsA at final concentrations of 5, 10, or 20 μmol/L are shown in Fig 5 and S10 Fig. Values are mean ± SEM normalized to basic (B) condition (one-way ANOVA, *P<0.05, 30 minutes exposure n = 3, 90 minutes exposure n = 4).

To study the acute effect of CsA on NCC abundance in kidney, mouse cortical tubules were isolated and exposed to CsA. All mouse cortical tubule suspensions were incubated in basic buffer for 30 or 90 minutes in the absence or presence of CsA (at final concentrations of 5, 10, and 20 μmol/L). No significant changes were observed in tNCC, while the pNCC abundance and the ratio of pNCC to tNCC were significantly increased after 30 minutes of exposure to 10 or 20 μmol/L of CsA, and after 90 minutes of exposure to 5, 10, or 20 μmol/L of CsA (Fig 5). Hypotonic low chloride, which decreases the intracellular chloride concentration thereby activating the WNK-SPAK and OSR1 pathways [41,42], was used as a positive control. pNCC and the ratio of pNCC to tNCC were significantly increased in hypotonic low chloride buffer, whereas tNCC remained unchanged (Fig 5).

## Discussion

Our study demonstrates that chronic treatment with CNIs increases both tNCC and pNCC abundance in uEVs of kidney transplant recipients, suggesting increased sodium reabsorption in the distal part of the nephron. Additionally, we show that the increase in pNCC abundance in uEVs of kidney transplant recipients correlates with the blood pressure response to NCC-inhibiting thiazide diuretics. Lastly, we corroborate that CNIs activate NCC in the kidney by showing that short-term exposure to cyclosporine increases pNCC but not tNCC abundance in mouse cortical tubules suspensions. Collectively, these observations indicate that the CNI-induced increase in NCC abundance and activity are involved in the pathogenesis of hypertension in kidney transplant recipients, and suggest that pNCC abundance in uEVs could be used as a biomarker to predict the blood pressure response to thiazide diuretics.

Since the introduction of CNIs in the 1980s, there has been a steep increase in the prevalence of hypertension after kidney transplantation [46,47]. In a study using a kidney specific 12 kDa FK506-binding protein, FKBP12, knockout mice, Tac caused hypertension by inhibiting calcineurin directly in DCT cells expressing NCC [22]. Moreover, NCC knockout mice were protected from tacrolimus-induced hypertension [21], confirming the role of NCC in CNI-induced hypertension. Although both CsA and Tac inhibit calcineurin, they differ in structure and cytoplasmic binding protein, which might explain why hypertension is less common and less severe in patients using Tac than in those using CsA [2,6–8,10,48]. However, it is still not known whether the higher incidence of hypertension in CsA- compared to Tac-treated kidney transplant recipients is due to a stronger tNCC and pNCC upregulation. Our study demonstrates that CsA does not increase tNCC and pNCC abundances in uEVs of kidney transplant recipients more than Tac (Fig 2). This suggests that factors other than NCC might contribute to the discrepancy in the increased blood pressure between patient groups using CsA and Tac. It is known that CNIs affect tissues other than the kidney, which are also involved in the development of hypertension [22]. CsA and Tac might differ in their ability to cause vasoconstriction [49,50], activate the renin-angiotensin-aldosterone system (RAAS) [21,50], or the sympathetic nervous system [51], all of which can contribute to the development of hypertension.

Several guidelines recommend thiazide-type diuretics as first-line treatment for the management of hypertension in adults [52,53]. Thiazide-type diuretics act by blocking NCC, thus increasing sodium excretion by the kidney [54]. Given the role of NCC in CNI-induced hypertension, thiazide diuretics might be especially effective drugs for hypertensive transplant recipients using CNIs and NCC abundance in their uEVs might predict the blood pressure response to thiazide diuretics. Indeed, uEV analysis in patients selected from our crossover study demonstrates that pre-treatment abundances of tNCC and pNCC in uEVs predict the blood pressure response to thiazide diuretics (Fig 3). This implies that the abundance of pNCC in uEVs of hypertensive kidney transplant recipients using CNIs could be used to predict thiazide sensitivity. Although it may be more pragmatic to simply test the response to a trial of thiazide diuretics, urinary biomarkers may help individualize anti-hypertensive treatment, especially with the development of high-throughput assays [55]. However, acute regulators of NCC activity such as the RAAS and potassium status should also be taken into account when interpreting the obtained results (see below). A relevant question is what explains the higher pNCC and tNCC in uEVs of responders prior to treatment, and the decrease in ratio of pNCC to tNCC in non-responders after thiazide treatment. One could argue that CNI-induced NCC activation was greater in responders. An alternative possibility is that the potassium balance determined thiazide sensitivity. A high potassium diet and hyperkalemia have recently been shown to inhibit NCC [56,57]. Indeed, serum potassium concentrations were higher in non-responders compared to responders. Here, we show that NCC abundance was increased in the majority of “responders” after thiazide treatment (Fig 4), which is consistent with data obtained in animal models [58,59]. Na *et al*. showed with immunoblot analysis and immunostaining that chronic hydrochlorothiazide treatment in rats increased the abundance of NCC [58]. Moreover, chronic hydrochlorothiazide infusion in mice increased the binding density of [^3^H] metolazone, an indirect measure of NCC activity, which confirms the increase of NCC after hydrochlorothiazide [60]. Similarly, chronic administration of the diuretics furosemide and amiloride (which blocks the Na-K-Cl cotransporter and ENaC, respectively), increased the abundance of these proteins in rodent kidney [58,61]. These effects might be the result of compensatory mechanisms, potentially mediated by the RAAS or/and potassium balance, which counteract reduced NCC function by the thiazide treatment [62]. Of interest, non-responders had a completely different pattern during thiazide treatment with a decrease in pNCC and ratio of pNCC to tNCC (Fig 4). The explanation for this difference is unclear, but one might speculate that a blood pressure response to thiazides is accompanied by RAAS activation resulting in different NCC excretion patterns in uEVs. Alternatively, differences in potassium balance may explain these results, because a recent study showed that pNCC stimulation by angiotensin II occurs as a compensatory response to renal potassium loss [63].

To further confirm the effect of CNIs on NCC in the kidney, we performed an *ex vivo* study using mice cortical tubules. This experiment demonstrated that short-term exposure to CsA increases pNCC abundance, while tNCC remained stable (Fig 5). This phenomenon was previously reported by Hoorn *et al*. [21] in mice and human embryonic kidney 293 cells treated with Tac, and by Melnikov *et al*. [23] in mDCT cells treated with CsA. Recently, it was demonstrated in mice that treatment with Tac prevents the acute high potassium induced NCC dephosphorylation, while tNCC remained unaffected [24]. In contrast to previous studies, this acute regulatory system mediated by calcineurin is shown to be independent of the WNK-SPAK signaling cascade [24]. Our study demonstrated that chronic administration of CNIs increases the abundance of both tNCC and pNCC in uEVs of kidney transplant recipients. This suggests that the difference in tNCC increase between mice cortical tubules and uEVs might be dependent on the time of stimulation or another signaling molecule involved in the *in vivo* situation.

A number of limitations of our study should be mentioned. First, several other factors, in addition to CNIs, may regulate NCC, which may also explain differences in expression of NCC and in thiazide sensitivity. Second, an unresolved question in the uEV-field remains whether the abundance of protein per uEV varies, or that the number of uEVs is regulated. Although we included the uEV-marker CD9, techniques that allow uEV counting will be necessary to address this point more conclusively.

## Conclusions

Our findings demonstrate that CNI treatment increases both tNCC and pNCC abundance in uEVs isolated from kidney transplant recipients. We also show that the blood pressure response to chlorthalidone in Tac-treated hypertensive kidney transplant recipients was related to the pNCC abundance in uEVs. This implies that pNCC in uEVs of kidney transplant recipients treated with a CNI might be used to predict blood pressure response to thiazide diuretics. In addition, uEV analysis may have clinical utility as a non-invasive biomarker for a variety of physiological and pathological conditions.

## Supporting information

S1 Excel Excel file show the optical densitometry of Fig 1 and S3 and S4 Figs. Both the dimeric and monomeric forms of the tNCC and pNCC bands on the blots were quantified together with Image Studio Lite software (LI-COR Biosciences, NE, USA).
(XLSX)

S2 Excel Excel file show the optical densitometry of Fig 5 and S10 Fig. tNCC and pNCC bands on the blots were quantified with Image Studio Lite software (LI-COR Biosciences, NE, USA).
(XLSX)

S3 Excel This excel file shows the optical densitometry data of Figs 3 and 4 and S5, S6 and S8 Figs. Both the dimeric and monomeric forms of the tNCC and pNCC bands on the blots were quantified together.
(XLSX)

S4 Excel This excel file shows the optical densitometry data of S7 Fig. It contains the data of tNCC and pNCC in responders compared to non-responders for both males and females separately.
(XLSX)

S5 Excel This excel file shows the optical densitometry data of S9 Fig. It contains the data of tNCC and pNCC normalized to CD9.
(XLSX)

S1 Fig Males versus females densitometry of tNCC and pNCC immunoreactive bands in uEVs of all kidney transplant recipients treated with CsA (male n = 5, female n = 4), Tac (male n = 7, female n = 6) or CNI-free immunosuppressive regimens (male n = 12, female n = 11) and healthy volunteers (male n = 6, female n = 0). Both in males and females tNCC (A and B) and pNCC (C and D) abundance in both CsA- and Tac-treated kidney transplant recipients was significantly higher in comparison to kidney transplant recipients treated with CNI-free immunosuppressive regimens. The ratio of pNCC to tNCC abundance in uEVs of CsA- and Tac-treated group was not significantly more abundant in comparison to kidney transplant recipients treated with CNI-free immunosuppressive regimens (E-F). The original immunoblots, are shown in Fig 1 and S3 and S4 Figs. Densitometry data are shown in S1 Excel. Values are mean ± SEM normalized to kidney transplant recipients treated with CNI-free immunosuppressive regimens (one-way ANOVA, *P<0.05, n = 51).
(TIF)

S2 Fig Densitometry of tNCC and pNCC immunoreactive bands in uEVs of all kidney transplant recipients treated with CsA (n = 9), Tac (n = 13) or CNI-free immunosuppressive regimens (n = 21) and healthy volunteers (n = 6). The volume of uEV suspension per sample was adjusted according to the urinary creatinine concentration and loaded on gels for immunoblot analysis. Densitometry analysis of the immunoblots for the abundance of tNCC (A) and pNCC (B). Both the dimer and monomer bands were analyzed together. The original immunoblots are shown in Fig 1 and S3 and S4 Figs. The abundance of both tNCC and pNCC was normalized to CD9 abundance after normalization by urinary creatinine. Densitometry data are shown in S1 Excel. Values are mean ± SEM normalized to kidney transplant recipients treated with CNI-free immunosuppressive regimens (one-way ANOVA, *P<0.05, n = 51).
(TIF)

S3 Fig Immunoblots of tNCC abundance in uEVs of kidney transplant recipients treated with CsA, Tac or CNI-free immunosuppressive regimens and healthy volunteers (A-D). uEV samples with a mark of # were loaded twice as a control on the gel, although these samples were excluded from densitometry analysis. Densitometry data are shown in S1 Excel.
(TIF)

S4 Fig Immunoblots of pNCC abundance in uEVs of kidney transplant recipients treated with CsA, Tac or CNI-free immunosuppressive regimens and healthy volunteers (A-C). uEV samples with a mark of # were loaded twice as a control on the gel, although these samples were excluded from densitometry analysis. Densitometry data are shown in S1 Excel.
(TIF)

S5 Fig This figure shows how Fig 4A was created. We used pNCC in uEVs before and after chlorthalidone treatment of responders (A) and non-responders (B).
(TIF)

S6 Fig This figure shows how Fig 4A was created. We used tNCC in uEVs before and after chlorthalidone treatment of responders (A) and non-responders (B).
(TIF)

S7 Fig This figure depicts the densitometry results of tNCC and pNCC comparable to Fig 3A and 3B, but separated into male and female. Panel A and C show tNCC and pNCC in males for responders (n = 6) compared to non-responders (n = 6). Panel B and D show tNCC and pNCC in females for responders (n = 4) compared to non-responders (n = 2). The non-parametric t-test was used for the analysis of all the graphs in this figure, *P<0.05.
(TIF)

S8 Fig This figure shows the immunoblots of CD9 abundance of Fig 4A and S5 and S6 Figs. The abundance of CD9 was comparable before and after chlorthalidone treatment of responders (A) and non-responders (B).
(TIF)

S9 Fig This figure depicts the densitometry results of tNCC and pNCC normalized to CD9. tNCC and pNCC normalized to CD9 abundance of both responders and non-responders that are depicted in panel A and B (n = 18 for both). Panel C and D show the data for tNCC and pNCC normalized to CD9 in responders before and after treatment with chlorthalidone (n = 20 for both). Panel E and F show the data for tNCC and pNCC normalized to CD9 in non-responders before and after treatment with chlorthalidone (n = 16 for both). The non-parametric t-test was used for the analysis of all the graphs in this figure, *P<0.05.
(TIF)

S10 Fig This figure shows the immunoblots tNCC and pNCC abundance in mouse cortical tubule suspension exposed to CsA. Immunoblots of protein homogenates of mouse cortical tubule suspensions were incubated in basic (B) buffer for 30 (A-C) and 90 minutes (C-F), in the absence (-) or presence of CsA at final concentrations of 5, 10, or 20 μmol/L. Densitometry data are shown in S2 Excel.
(TIF)
