# Supplementary figures and images for: NaCl cotransporter abundance in urinary vesicles is increased by calcineurin inhibitors and predicts thiazide sensitivity
Source: PLoS One. 2017 Apr 21;12(4):e0176220. doi: 10.1371/journal.pone.0176220 (PMC5400280; doi:10.1371/journal.pone.0176220)

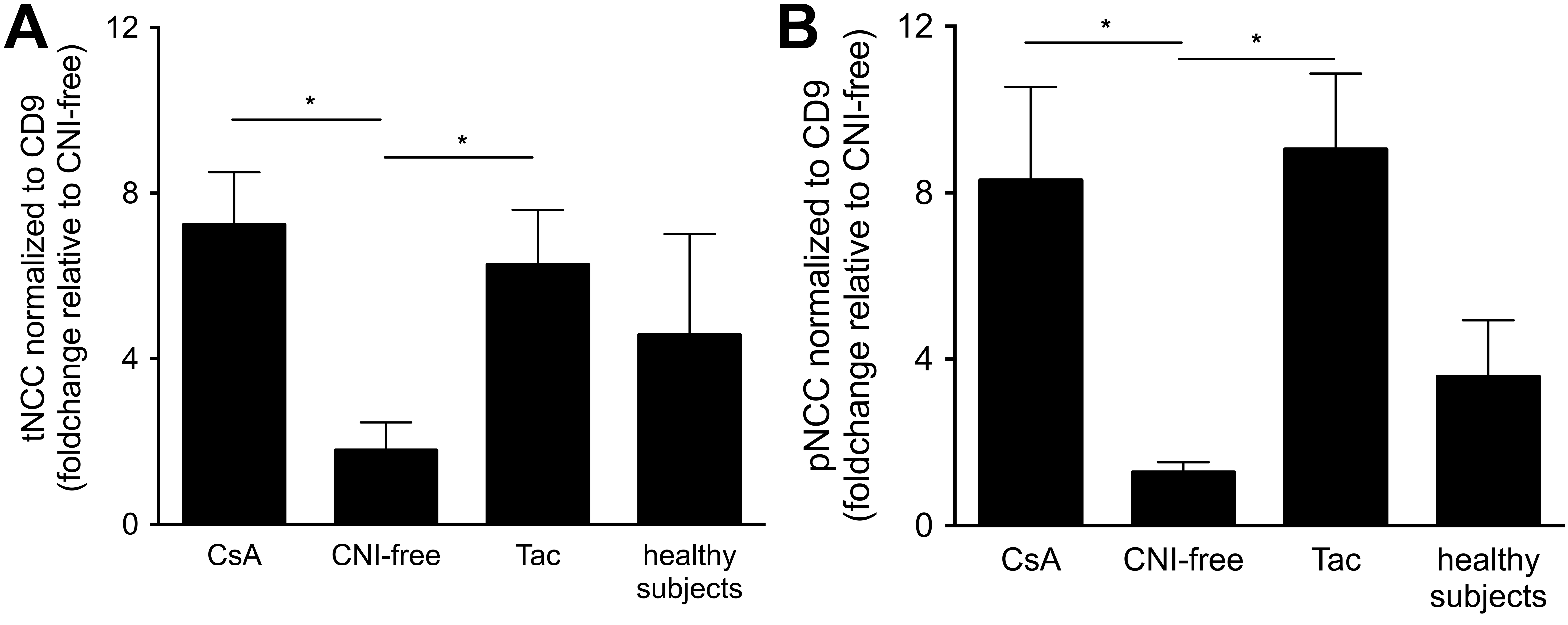

Supplement: S2 Fig — The volume of uEV suspension per sample was adjusted according to the urinary creatinine concentration and loaded on gels for immunoblot analysis. Densitometry analysis of the immunoblots for the abundance of tNCC (A) and pNCC (B). Both the dimer and monomer bands were analyzed together. The original immunoblots are shown in Fig 1 and S3 and S4 Figs. The abundance of both tNCC and pNCC was normalized to CD9 abundance after normalization by urinary creatinine. Densitometry data are shown in S1 Excel. Values are mean ± SEM normalized to kidney transplant recipients treated with CNI-free immunosuppressive regimens (one-way ANOVA, *P<0.05, n = 51). (TIF) [file pone.0176220.s007.tif]

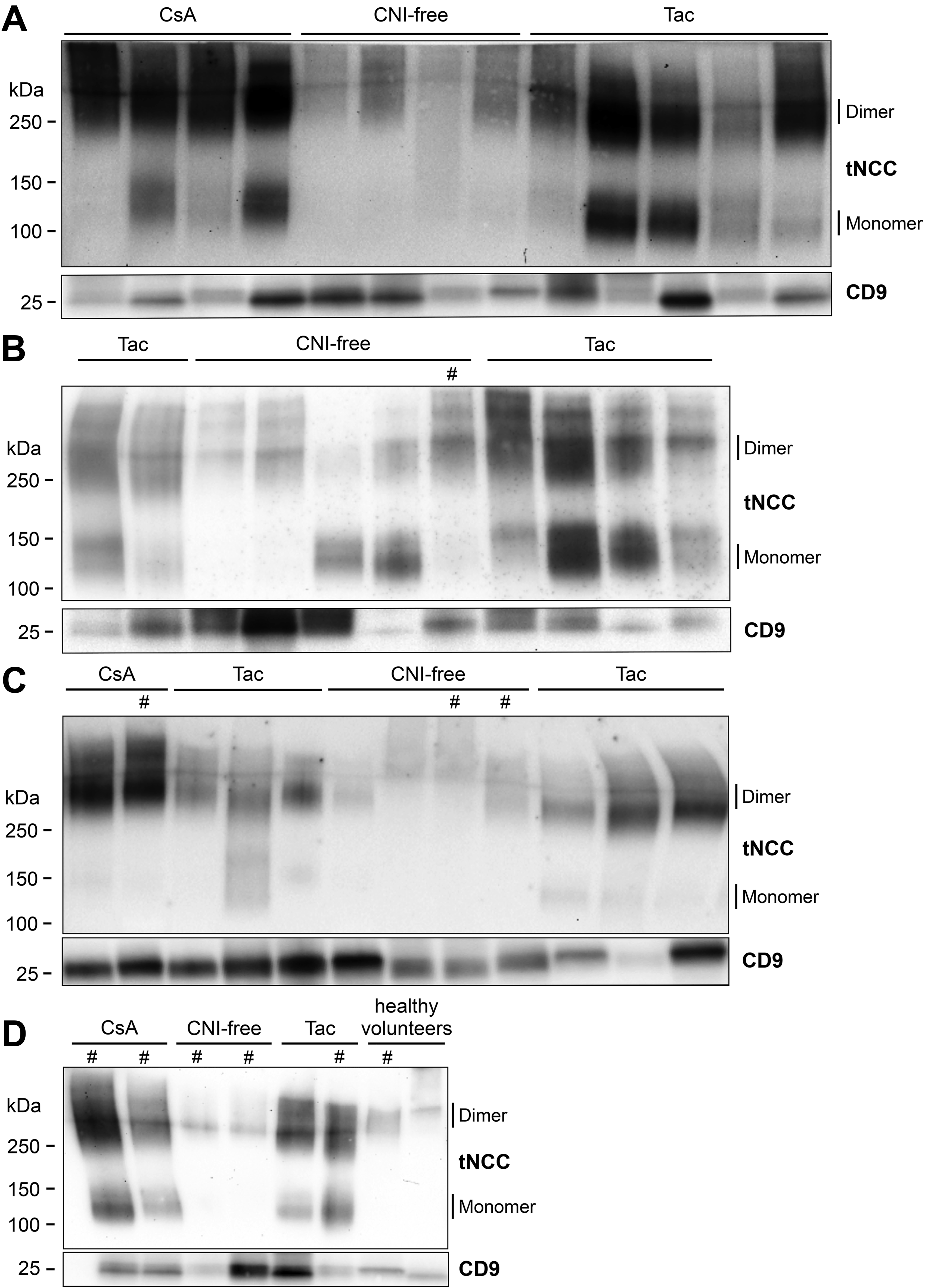

Supplement: S3 Fig — uEV samples with a mark of # were loaded twice as a control on the gel, although these samples were excluded from densitometry analysis. Densitometry data are shown in S1 Excel. (TIF) [file pone.0176220.s008.tif]

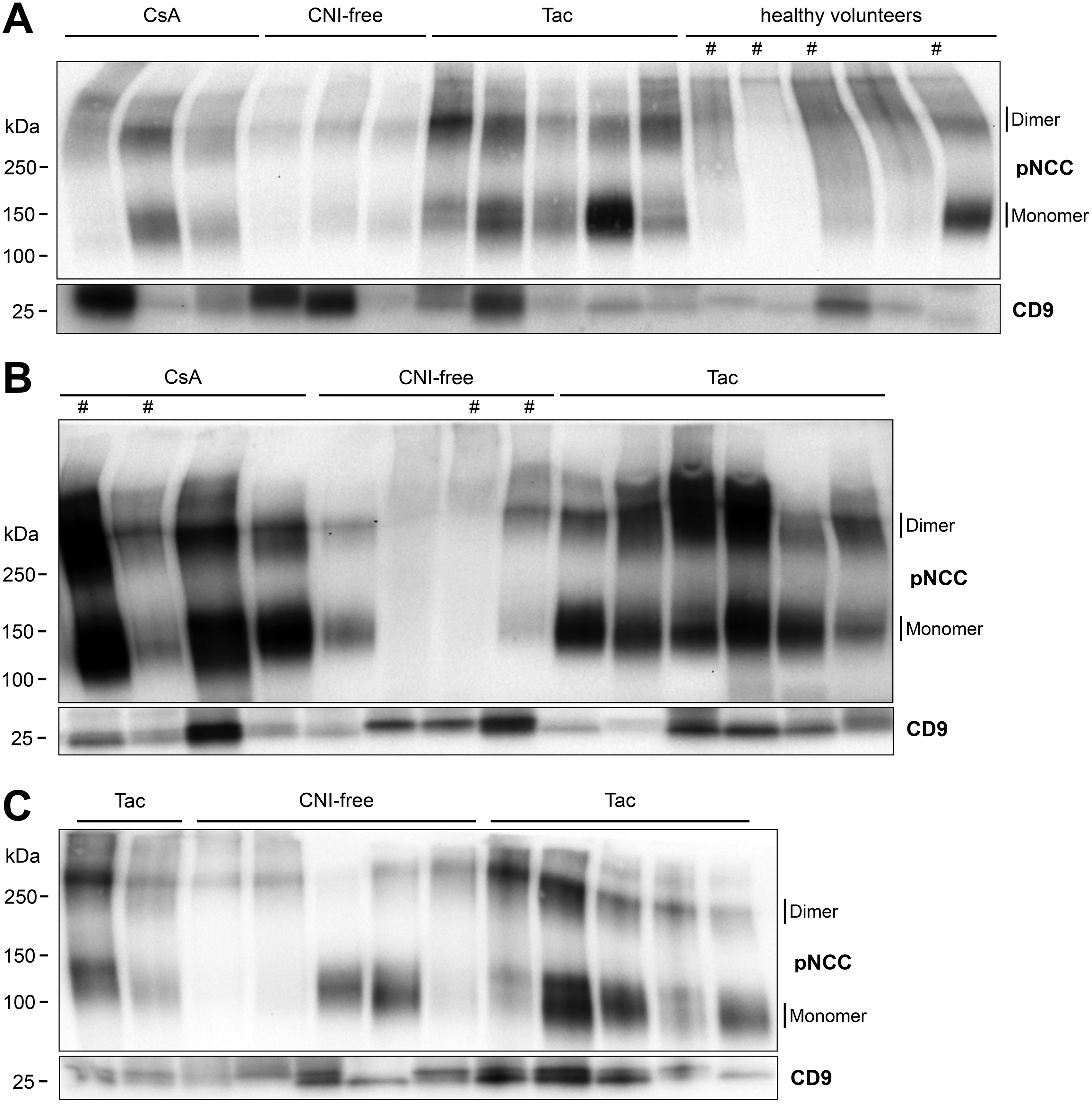

Supplement: S4 Fig — uEV samples with a mark of # were loaded twice as a control on the gel, although these samples were excluded from densitometry analysis. Densitometry data are shown in S1 Excel. (TIF) [file pone.0176220.s009.tif]

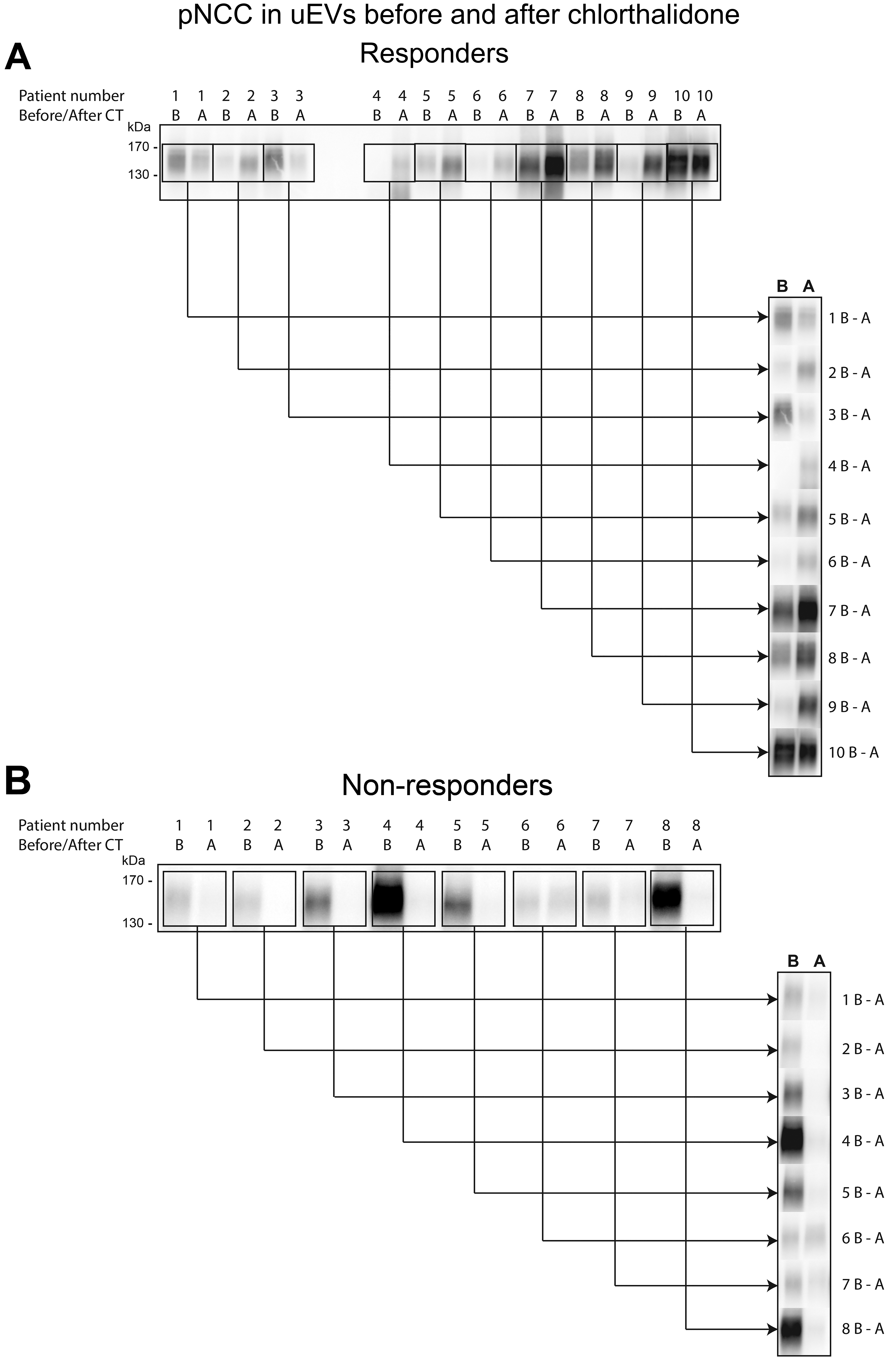

Supplement: S5 Fig — We used pNCC in uEVs before and after chlorthalidone treatment of responders (A) and non-responders (B). (TIF) [file pone.0176220.s010.tif]

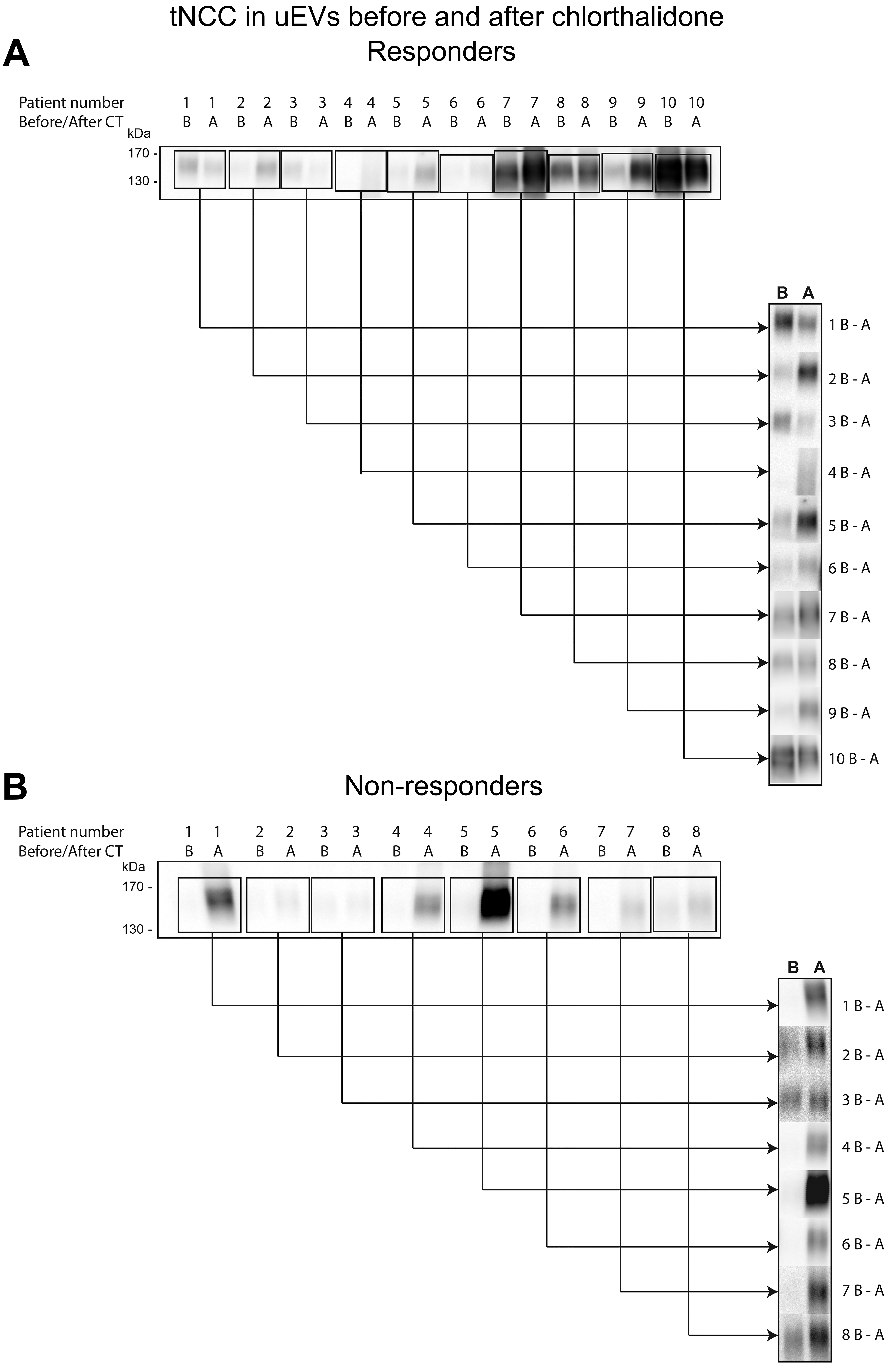

Supplement: S6 Fig — We used tNCC in uEVs before and after chlorthalidone treatment of responders (A) and non-responders (B). (TIF) [file pone.0176220.s011.tif]

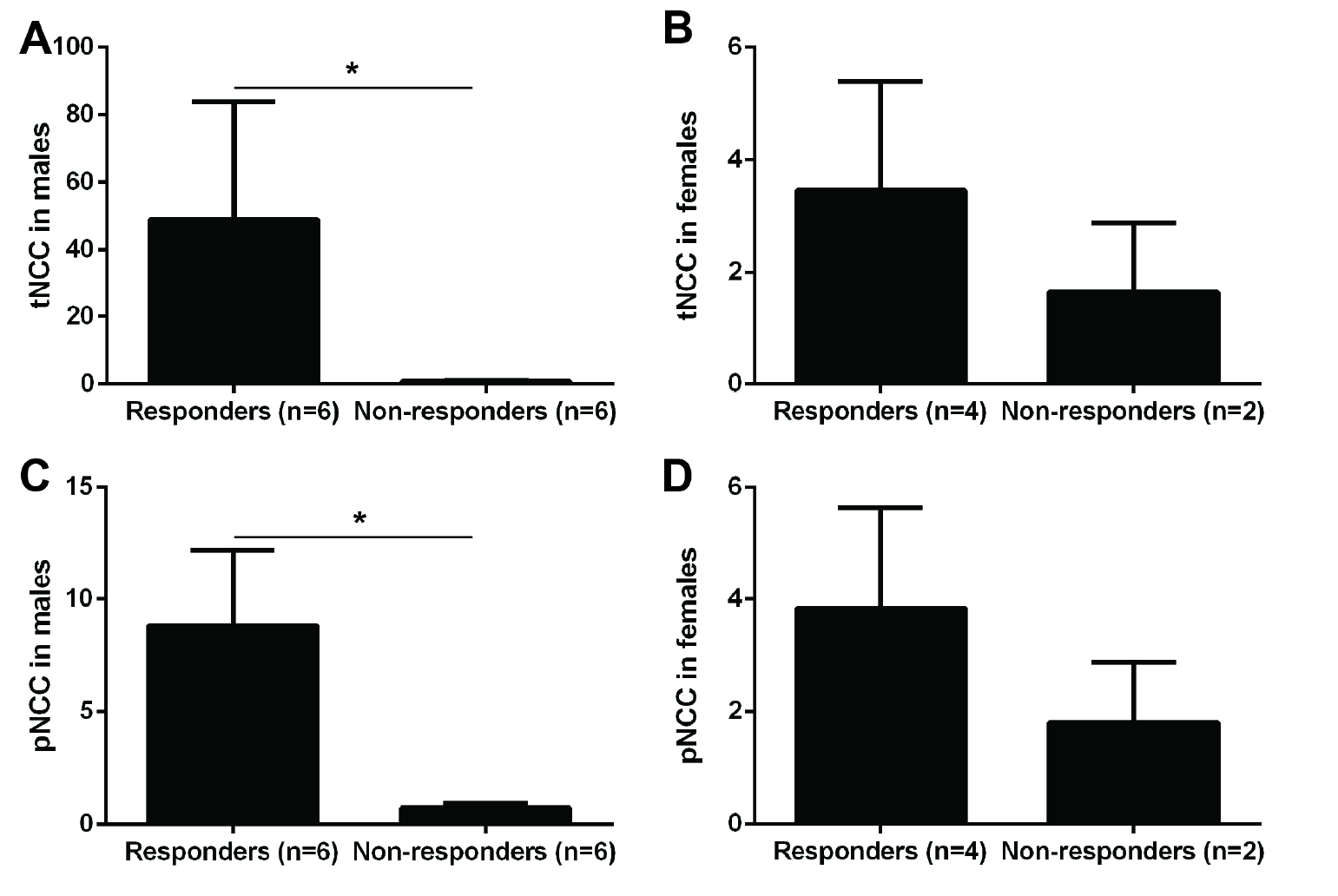

Supplement: S7 Fig — Panel A and C show tNCC and pNCC in males for responders (n = 6) compared to non-responders (n = 6). Panel B and D show tNCC and pNCC in females for responders (n = 4) compared to non-responders (n = 2). The non-parametric t-test was used for the analysis of all the graphs in this figure, *P<0.05. (TIF) [file pone.0176220.s012.tif]

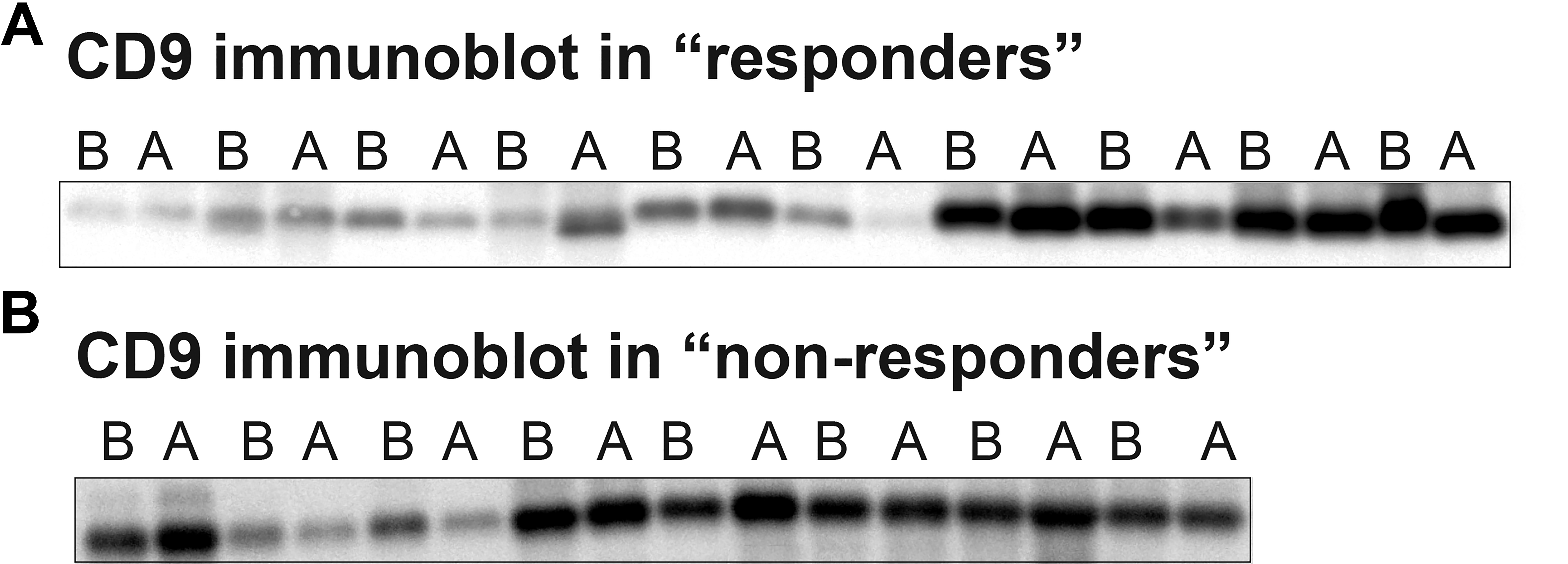

Supplement: S8 Fig — The abundance of CD9 was comparable before and after chlorthalidone treatment of responders (A) and non-responders (B). (TIF) [file pone.0176220.s013.tif]

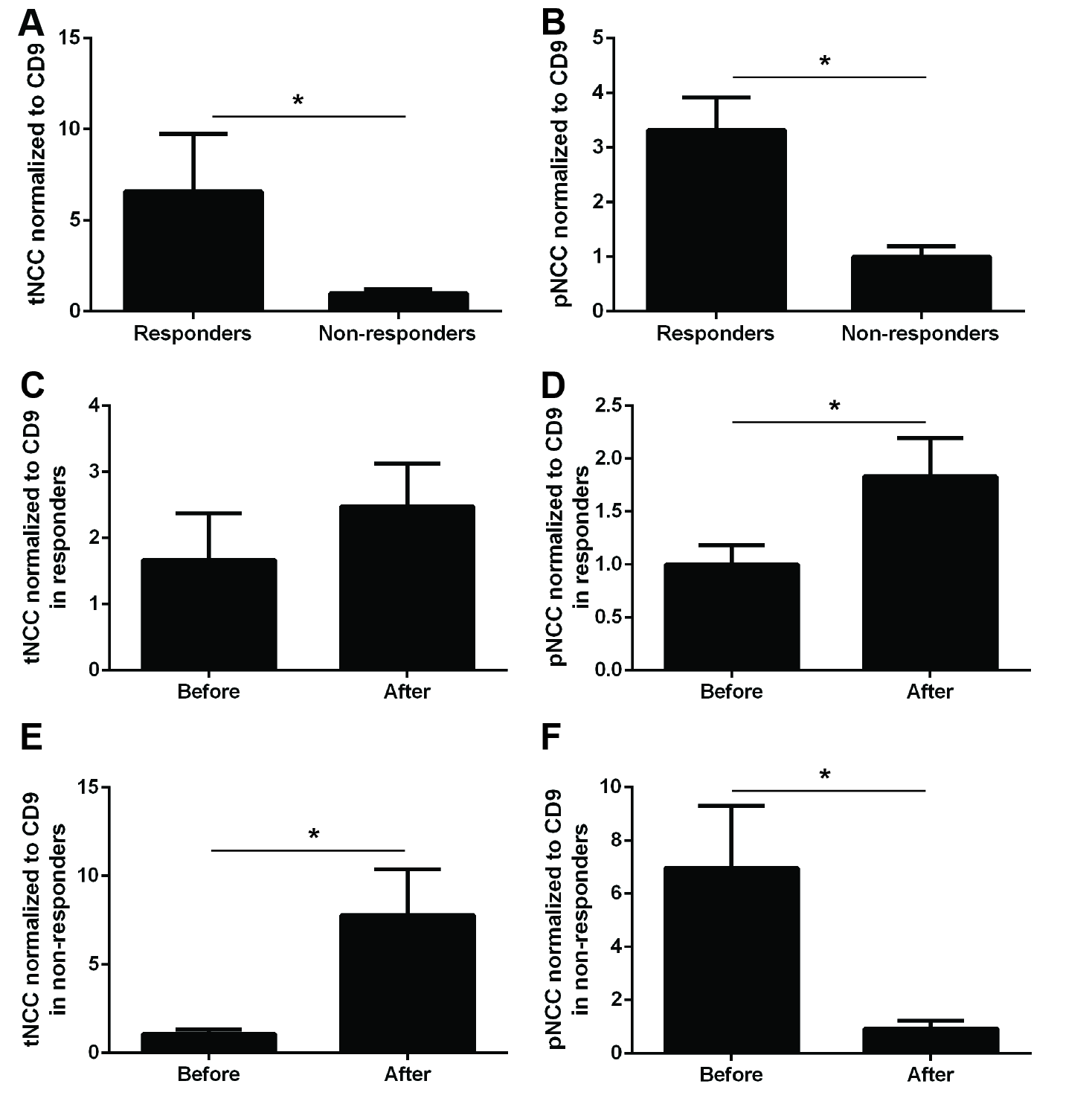

Supplement: S9 Fig — tNCC and pNCC normalized to CD9 abundance of both responders and non-responders that are depicted in panel A and B (n = 18 for both). Panel C and D show the data for tNCC and pNCC normalized to CD9 in responders before and after treatment with chlorthalidone (n = 20 for both). Panel E and F show the data for tNCC and pNCC normalized to CD9 in non-responders before and after treatment with chlorthalidone (n = 16 for both). The non-parametric t-test was used for the analysis of all the graphs in this figure, *P<0.05. (TIF) [file pone.0176220.s014.tif]

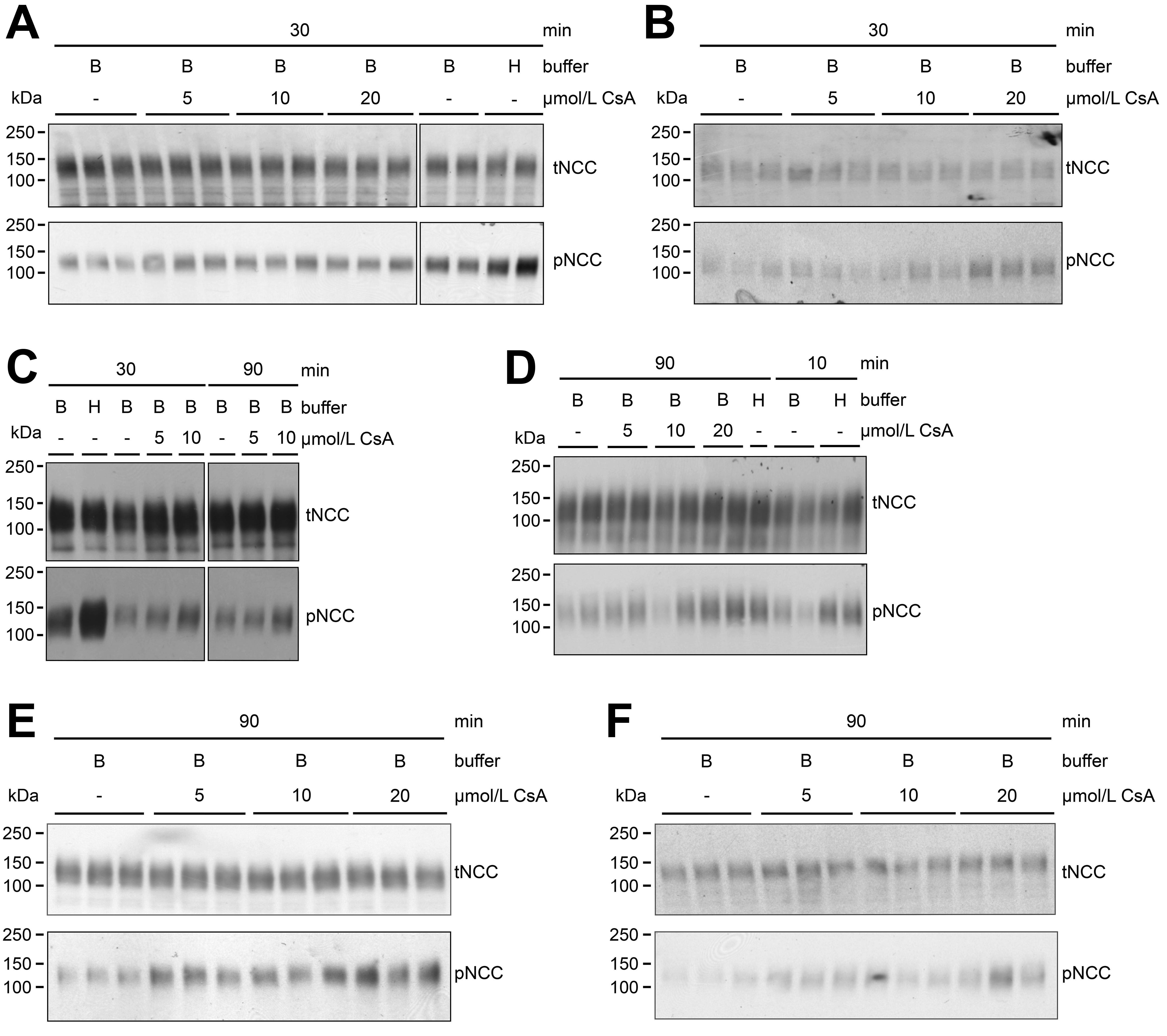

Supplement: S10 Fig — Immunoblots of protein homogenates of mouse cortical tubule suspensions were incubated in basic (B) buffer for 30 (A-C) and 90 minutes (C-F), in the absence (-) or presence of CsA at final concentrations of 5, 10, or 20 μmol/L. Densitometry data are shown in S2 Excel. (TIF) [file pone.0176220.s015.tif]
